# Use of the AFX Stent Graft in Patients with Extremely Narrow Aortic Bifurcation: A Multicenter Retrospective Study

**DOI:** 10.1155/2021/7439173

**Published:** 2021-10-04

**Authors:** M. U. Wagenhäuser, N. Floros, E. Nikitina, J. Mulorz, K. M. Balzer, S. Goulas, M. Petrich, P. Dueppers, F. Simon, H. Schelzig, A. Oberhuber

**Affiliations:** ^1^Department of Vascular and Endovascular Surgery, Medical Faculty and University Hospital Duesseldorf, Heinrich-Heine-University, Moorenstraße 5, 40225 Duesseldorf, Germany; ^2^Department of Anesthesiology, Medical Faculty and University Hospital Duesseldorf, Heinrich-Heine-University, Moorenstraße 5, 40225 Duesseldorf, Germany; ^3^Department of Vascular and Endovascular Surgery, St. Marien-Hospital Bonn, Robert-Koch-Straße 1, 53115 Bonn, Germany; ^4^Department of Vascular and Endovascular Surgery, Hubertus Protestant Hospital Berlin, Spanische Allee 10-14, 14129 Berlin, Germany; ^5^Department of Vascular and Endovascular Surgery, University Hospital Muenster, Westphalian Wilhelm University, Albert-Schweitzer-Campus 1, 48149 Muenster, Germany

## Abstract

**Introduction:**

This study analyzed the patient outcomes following endovascular aortic aneurysm repair (EVAR) for infrarenal aortic pathologies with very narrow aortic bifurcations using the AFX stent graft.

**Methods:**

The data was retrieved from the archived medical records of 35 patients treated for abdominal aortic aneurysm (AAA) (48.6%) or penetrating aortic ulcer (PAU) (51.4%) with very narrow aortic bifurcation between January 2013 and May 2020. Patient survival, freedom from endoleak (EL), and limb occlusion were estimated applying the Kaplan–Meier method.

**Results:**

The mean follow-up time was 20.4 ± 22.8 months. The mean aortic bifurcation diameter was 15.8 ± 2.2 mm. Technical success was 100%, and no procedure-related deaths occurred. Two type II ELs occurred within 30-day follow-up. We observed one common iliac artery stenosis at four months and one type III EL at 54 months in the same patient, both of which required re-intervention. Overall patient survival was 95 ± 5% (AAA: 100%; PAU: 89 ± 10%), freedom from limb occlusion was 94 ± 5% (AAA: 91 ± 9%; PAU: 100%), freedom from type II EL was 94 ± 4% (AAA: 88 ± 8%; PAU: 100%), and freedom from EL type III was 83 ± 15% (AAA: 80 ± 18%; PAU: 100%) at the end of the follow-up period.

**Conclusions:**

Very narrow aortic bifurcations may predispose patients to procedure-related complications following EVAR. Our results suggest a safe use of the AFX stent graft in such scenarios. The overall short- and long-term procedure-related patient outcomes are satisfying albeit they may seem superior for PAU when compared to AAA.

## 1. Introduction

EVAR has evolved as first-line treatment for nonruptured abdominal aortic aneurysm (AAA) and or penetrating aortic ulcer (PAU) since the early 2000s. Due to superior outcomes in regard to in-hospital morbidity and mortality, EVAR has surpassed open surgical repair (OR) in popularity in most countries [1, 2]. Recent guidelines also recommend its use in ruptured AAA as the first-line approach [3].

Despite its favorable characteristics, EVAR should be performed within well-defined anatomical dimensions to achieve high technical success. Therefore, stent graft manufacturers have defined specific instructions for use (IFU), which physicians may follow to generate the greatest possible patient benefit [4]. Although the overall outside-of-IFU implantation rate may exceed 40% of all cases, it was not found to have worse aneurysm-related outcomes than treatment within the IFU [5, 6].

The aortic bifurcation remains a major location of concern. Here, a narrow bifurcation with a diameter < 20 mm is considered an anatomically unfavorable configuration that limits the application range for standard EVAR [7]. Such challenging aortic dimensions not only complicate sizing and endovascular case planning but may also require adjunctive procedures, which carry significant procedure-related risks. As a consequence, narrow aortic bifurcations of <20 mm are reported to be an independent risk factor for complications such as limb occlusions [8, 9]. Here, unibody stent grafts are considered to specifically address such clinical issues.

**Figure 1:**
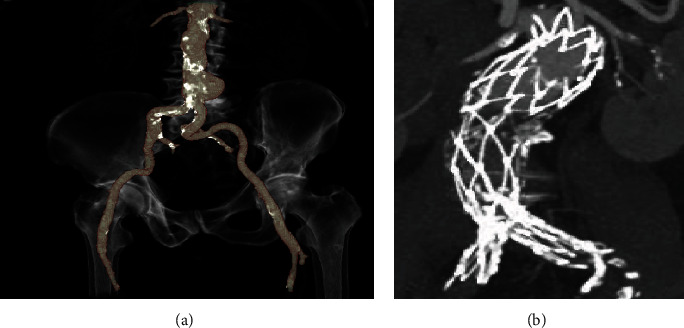
(a) Three-dimensional volume rendering of a penetrating aortic ulcer (PAU). The image illustrates an infrarenal PAU with a calcified and narrow aortic bifurcation prior to endovascular treatment. (b) Coronal multiplanar reformation following AFX stent graft placement at 3-year follow-up. The main unibody was deployed directly onto the aortic bifurcation. The proximal aortic tube stent graft extension seals in the infrarenal segment. No endoleak (EL), stent graft migration, or limb stenosis occurred after implantation.

The AFX stent graft (Endologix, Irvine, CA, USA) is a bifurcated unibody aortic stent graft that specifically addresses the issue of narrow aortic bifurcations. Conventional bifurcated endografts expose iliac limbs to competition in the narrow lumen and consequently increase the risk of stenosis or thrombosis. In contrast, the AFX stent graft aims a fixation directly onto the aortic bifurcation preventing limb competition in the distal aorta which may be particularly valuable in narrow aortic bifurcations. Further, the AFX stent graft allows for flexibility and optimal apposition of the polytetrafluoroethylene (PTFE) material, since the interconnected stents are attached only at the proximal and distal ends of the inner surface. The proximal expanded polytetrafluoroethylene- (ePTFE-) based aortic extension allows for infrarenal or suprarenal anatomical fixation [10, 11] **(**Figure 1**)**.

Recent studies suggestsafe and effective use of a unibody stent graft in aortic bifurcation diameters ranging from 17 to 20 mm [12, 13]. As of today, there is a paucity in current literature regarding the use of the AFX stent in even smaller aortic bifurcations. The present retrospective multicenter study analyzed the patient outcome using the AFX stent graft for AAA or PAU with very narrow aortic bifurcations at an average of 15.6 mm.

## 2. Material and Methods

The study was conducted at four German centers. The study was approved by the ethic committee of the medical faculty of the Heinrich-Heine-University Düsseldorf, Germany (study number 6117R). Patient data was collected in retrospect for patients treated between January 2013 and May 2020. Inclusion criteria were aortic bifurcation ≤ 18 mm, AAA, or PAU mandating treatment according to current guidelines, usage of the AFX stent graft, and age ≥ 18 years. Major adverse events were reported following the major adverse cardiac and cerebrovascular events (MACCE) classification [14].

During the study period, more than 1000 EVAR procedures were performed across the participating centers. Of these, the AFX stent graft was used in 53 cases. We included 35 patients treated with the AFX stent graft for AAA or PAU who met the aforementioned inclusion criteria. Patient's medical records were screened for demographics, disease-specific risk factors, medication, reports of physical examinations, surgery reports, and computed tomography angiography (CTA) scans. Patients underwent their first follow-up examination within 30 days following surgery by contrast-enhanced ultrasound (CEUS) or CTA scans to screen for stent graft-related complications. Thereafter, patients were planned for yearly follow-up examinations. OsiriX MD version 10.0 was used to measure angulation-adjusted aortic dimensions. The proportion of aortic circumference covered with calcification was evaluated and stratified into four groups (1: no calcification; 2: <1/3; 3: 1/3-2/3; and 4: full circumference) [15]. The presence of thrombus burden in AAA or PAU and iliac kinking was further analyzed.

Statistical analysis was performed using SPSS 17.0 for Windows (SPSS Inc., Chicago, IL). Kaplan–Meier estimator was applied to calculate patient survival, freedom from endoleak (EL), and limb stenosis. Kaplan–Meier estimations are presented as mean ± standard error, and the log-rank test was used to analyze differences between PAU and AAA patients. Descriptive statistics are reported as mean ± standard deviation or relative frequencies with percentages. Student's *t*-test or Mann–Whitney *U* test was applied according to the Kolmogorov-Smirnov normality test to analyze differences between AAA and PAU patients for key morphological and procedural parameters.

## 3. Results

### 3.1. Patient Characteristics

The mean patient age was 73.8 ± 7.2 years. The study included a total of 35 patients of which 17 patients (48.6%) were treated for AAA and 18 patients (51.4%) for PAU. Patient baseline characteristics were similar between AAA and PAU patients (Table 1**)**. Further, one patient (2.9%) required AFX stent graft placement for type Ib EL following tube stent graft placement (Medtronic, Dublin, Ireland) for PAU with an insufficient distal landing zone. The overall mean maximum aortic diameter prior to AFX stent graft implantation was 44.0 ± 11.4 mm, and the overall mean aortic bifurcation diameter was 15.8 ± 2.2 mm. The infrarenal aortic “neck” diameter/length and the maximal aortic diameter were bigger for AAA patients when compared to PAU patients (*p* < 0.05). All other key morphological parameters were similar for AAA and PAU patients (Table 2). The proportion of aortic circumference covered with calcification was as follows: group 1: 1 (2.9%), group 2: 20 (57.1%), group 3: 12 (34.3%), and group 4: 2 (5.7%). We observed 19 patients (54.3%) with significant thrombus burden and 15 patients (42.9%) with kinking of the iliac arteries.

When considering antiplatelet therapy and/or anticoagulation, there was information available for 33 patients. There were 18 patients (51.4%) who were given single antiplatelet therapy (SAPT). Of these, 17 patients (48.6%) received treatment with aspirin while one patient (2.9%) received clopidogrel. Further, 8 patients (22.9%) received dual antiplatelet therapy (DAPT). Oral anticoagulation was given to 7 patients (20%), 4 of them in combination with aspirin (Table 1).

### 3.2. Procedural Parameters

There were 32 (91.4%) procedures performed under general anesthesia and 3 (8.6%) under local anesthesia. Surgical cut-down was necessary in 19 (54.3%) procedures, while percutaneous access was used in 16 (45.7%) cases. The overall mean procedure time was 114.8 ± 39.9 min. All key procedural parameters such as the total procedure time, the fluoroscopy time, and the volume of contrast agent were shorter/less for PAU patients when compared to AAA patients (*p* < 0.05) (Table 3). The mean component overlap was 48.3 ± 11.5 mm (AAA: 48.3 ± 11.5 mm; PAU: 48.5 ± 11.1 mm). Of note, the mean component overlap increased during the study period from 46.3 ± 11.1 mm in 2013 to 51.8 ± 7.4 mm in 2019. There were 12 AFX deployments without proximal aortic extension (AAA: 4; PAU: 8). No perioperative deaths, graft thrombosis, intraoperative conversion, stent graft migration, or ELs on the final angiograms were observed.

We performed adjunctive procedures in 4 patients (11.4%) which included endarterectomy of the common femoral artery (CFA) in one PAU and AAA patient each (5.7%), iliac relining with bare metal stent (BMS) placement in one PAU patient (2.9%), and balloon angioplasty of the external iliac artery (EIA) and common iliac artery (CIA) in another AAA patient (2.9%). Given the standardized length and diameter for both iliac sides in the AFX stent grafts, the authors report one AAA patient (2.9%) with necessity for iliac extension.

### 3.3. 30-Day Follow-Up

Mean intensive care unit and in-hospital stays were 0.9 ± 0.9 days (AAA: 1.1 ± 0.9 days; PAU: 0.8 ± 0.4 days) and 8.2 ± 4.2 days (AAA: 8.7 ± 3.6 days; PAU: 7.5 ± 3.4 days), respectively.

We observed no deaths, reinterventions, or access-related pseudoaneurysm during the 30-day follow-up. We report 4 patients (11.4%) with a major adverse event according to the MACCE classification.

In detail, one PAU patient (2.9%), who had a history of intravenous drug abuse and HIV infection, suffered from contrast agent-associated acute kidney injury (AKI), requiring temporary hemodialysis. Due to consecutive respiratory decompensation and acute lung injury, the patient was transferred to a specialized weaning clinic. Further, 2 AAA patients (5.7%) suffered from acute coronary syndrome (ACS) with myocardial infarction, which required percutaneous coronary intervention, and one AAA patient (2.9%) presented with a new onset of atrial fibrillation, which was successfully converted to sinus rhythm using antiarrhythmic drugs.

There were 33 patients (94.3%, excluding patients with AKI and ACS) who underwent imaging at 30 days of follow-up. Of these, 20 patients (60.6%) received CTA scans, and 13 patients (39.4%) CEUS. Here, we observed a type II EL in 2 AAA patients (5.7%), which were treated conservatively. For these 2 AAA patients, we observed no AAA diameter progression during the complete follow-up nor AFX stent graft migration or limb occlusion.

### 3.4. Long-Term Follow-Up

**Figure 2:**
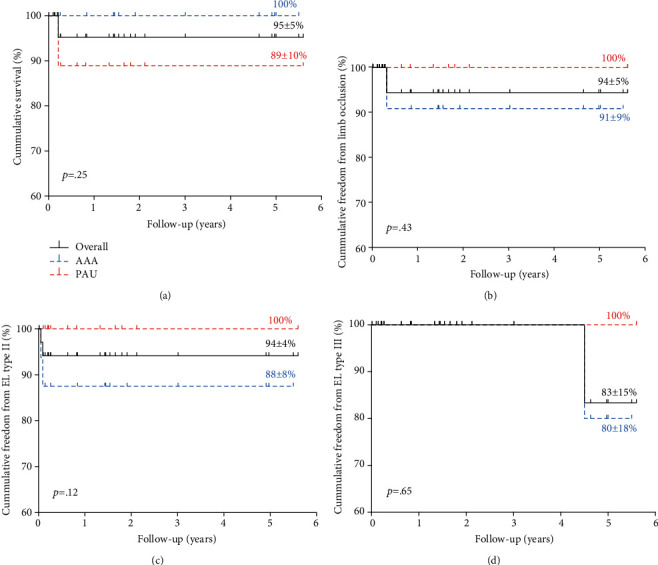
Kaplan–Meier estimator. Kaplan–Meier estimator with mean ± standard deviation for patient survival (a), freedom from limb occlusion (b), and freedom from type II (c) and type III (d) endoleak (EL). Patient survival was 95 ± 5%, freedom from limb occlusion was 94 ± 5%, "freedom from type II EL was 94 ± 4%, and freedom from type III EL was 83 ± 15% at the end of the follow-up after 5.6 years. The log-rank test was used to analyze differences between penetrating aortic ulcer (PAU) and abdominal aortic aneurysm (AAA) patients (*n* = 35).

The mean follow-up time was 20.4 ± 22.8 months (AAA: 23.6 ± 21.6 months; PAU: 18.5 ± 15.4 months). During this period, one death occurred in a PAU patient (2.9%) at two-month follow-up due to AKI on chronic kidney injury (CKI) and a hospital-acquired pneumonia. Patient survival was 95 ± 5% (AAA: 89 ± 10%; PAU: 100%) at the end of the follow-up period (Figure 2(a)). Notably, we observed a reduced mean maximum aortic diameter after AFX stent graft implantation when compared to the baseline (44 ± 11.4 mm vs. 40.7 ± 9‐9 mm) at 15.5 ± 12.8-month follow-up (AAA: 51.6 ± 9.5 mm vs. 48.7 ± 7.7 mm at 17 ± 15.8-month follow-up; PAU: 36.1 ± 6.8 mm vs. 33.8 ± 7.8 mm at 13.6 ± 10.6-month follow-up).

One AAA patient (2.9%) presented with limb occlusion at four-month follow-up. The patient had a history of peripheral arterial occlusive disease (PAOD) and was successfully treated with bilateral BMS implantation into the CIAs and balloon angioplasty of the right popliteal artery. At the end of the follow-up, overall freedom from limb occlusion was 94 ± 5% (AAA: 91 ± 9%; PAU: 100%) (Figure 2(b)).

Apart from the two type II ELs observed within 30 days poststent graft implantation, no additional type II EL was observed. Overall freedom from type II EL was 94 ± 4% (AAA: 88 ± 8%; PAU : 100%) at the end of the follow-up period (Figure 2(c)). One type III EL occurred at 54 months of follow-up in an AAA patient (2.9%) due to modular disconnection which required reintervention with implantation of 2 cuffs (diameter: 28 mm; length: 70 and 82 mm, Medtronic, Dublin, Ireland) into the AFX stent graft. The patient remained free from EL and did not require further reinterventions during the further follow-up. Consequently, freedom from type III EL was 100% after three years and 83 ± 15% (AAA: 80 ± 18%, PAU: 100%) at the end of the follow-up period (Figure 2(d)). Notably, we did not observe AAA or PAU rupture or stent graft migration in the study cohort, while limb occlusions and ELs only occurred in AAA patients. Nevertheless, our results did not find a significant difference between AAA and PAU patients regarding relevant patient outcomes at the long-term.

## 4. Discussion

This is the first study exclusively evaluating patients undergoing EVAR using the AFX stent graft for AAA or PAU with very narrow aortic bifurcations at a mean aortic bifurcation diameter of 15.8 mm. At the end of the follow-up period, overall patient survival was 95%, freedom from limb occlusion was 94%, and freedom from type II and III EL was 91% and 83%, respectively. Our data may advocate that these averaged patient outcomes may be superior for PAU patients when compared to AAA patients.

Given the lower 30-day mortality, morbidity, and shorter hospitalization time when compared to OR, EVAR has evolved as first-line treatment for infrarenal aortic pathologies [16]. However, in the long-term, EVAR requires subsequent surgery and/or re-interventions more frequently, suggesting the necessity of stringent follow-up to adequately detect stent graft-related complications [17, 18]. Morphology-related limitations of EVAR with bifurcated stent grafts are determined by the patient's anatomy and dimensions of the proximal and distal sealing zones [19]. Therefore, a distal aortic diameter of ≤20 mm may be considered a relative contraindication for bifurcated stent graft placement according to manufacturers' IFU [20]. Major concerns for EVAR in narrow distal aorta diameters arise from potential of vessel rupture, stent graft collapse, and occlusion. As a consequence, these pathologies are traditionally treated with aortoiliac stent grafts and femoral crossover bypass [21]. Although this approach is generally considered safe, patients are exposed to a considerable risk of graft infection [22]. Bifurcated stent grafts were introduced to overcome this issue by allowing for complete endovascular treatment with solid outcomes in terms of safety and patency [23].

The design of the AFX stent graft follows a unique concept, which aims for anatomical fixation onto the aortic bifurcation. By doing so, there are no competing limbs in the distal aorta, which may be advantageous in narrow anatomies and minimizes the risk of limb occlusion. The pillar-like design of the AFX stent graft allows for the placement of a proximal tube stent graft with infra- or suprarenal fixation [24]. In contrast to traditional bifurcated stent grafts, in which the limbs pass the aortic bifurcation parallelly, the AFX main unibody is deployed directly onto the aortic bifurcation, thus relining the distal aorta and CIAs [25].

Despite rapidly expanding experience with endovascular procedures, ELs remain the most frequent complication following EVAR for AAA and/or PAU [26]. Whereas type I and III ELs require urgent treatment, continued clinical surveillance is recommended for patients with type II EL and stable AAA diameter [27]. Notably, Gelfand et al. reported a spontaneous occlusion rate of up to 58% for type II ELs within one year [28]. This is also reflected in our results given that neither of the two patients with type II EL required re-intervention. The type II EL incidence is comparable to reports by other researchers using the same stent graft system. For example, Kouvelos et al. reported a type II EL rate of 10% after a median follow-up of 23 months when using the AFX stent graft, whereas Welborn et al. found a rate of 5.7% at 9-month follow-up [29, 30].

The authors are aware of the class I recall of July 2018 on all AFX stent graft systems for AAA due to a higher risk of type III EL. To address these concerns, the ultimately revised instructions for use recommend maximizing the overlap between the main unibody and proximal endoprosthesis component to eliminate modular disconnection and type III ELs. Accordingly, the authors observed an increase in mean component overlap during the study period, albeit the present study did not find an increased incidence of type II and III endoleaks when compared to the current literature. In addition, the reported EL rates of the present study using the AFX stent graft are comparable to those of recent studies using other stent graft devices in similar pathologies. For instance, Veraldi et al. reported a combined type I–III EL rate of 3.7% after a mean follow-up of 40 months in the narrow aortic bifurcation subgroup using the Gore Excluder (WL Gore & Associates, Inc., Newark, DE, USA) in a multicenter trial [31]. Notably, we observed 100% freedom from type III EL after the same follow-up period, albeit the mean aortic bifurcation diameter was 1.5 mm narrower. Our data further suggest that the risk for limb occlusion and type II and II ELs during the follow-up may be higher in AAA when compared to PAU. Notably, there is a large multicentric trial under way which will provide valuable mid- and long-term outcomes when using the AFX 2 stent graft [10].

Beyond ELs, limb occlusions are the most frequent cause of redo surgery and/or re-interventions following EVAR with an incidence of 0–7.2% [32]. Severe calcifications yield high intra-aortic radial forces and may predispose to limb occlusion following stent graft implantation [22]. In addition, tortuosity and significant angulation are well-established risk factors [33]. From a pathophysiological standpoint, a narrow distal aorta with severe calcifications may lead to significant differences in limb diameters, which in turn may favor limb occlusions. To underline the relevance of this concept, we report one case of limb occlusion in a patient with significant circular calcification of the aortic bifurcation and additional thrombus burden. Of note, the iliac artery diameter differences found in our study are comparable to those reported in other studies, indicating valid comparability of the reported outcomes [31, 34].

We report 94% long-term freedom from limb stenosis. Regarding this endpoint, Inaba et al. claimed a limb occlusion rate of 4% after a mean follow-up period of 37.1 months in 227 consecutive patients using various stent graft systems [32]. Interestingly, Melas et al. and Welborn et al. both reported no limb occlusions in their studies using the AFX stent graft [30, 35]. Considering these experiences, the AFX stent graft provides comparable, if not superior, freedom from limb occlusion in even narrower aortic bifurcations. Based on our experience, we may consider the AFX stent graft a reliable treatment option for these challenging infrarenal aortic pathologies.

Our study has several limitations. First, there was no rigid protocol for patient recruitment, treatment, or follow-up due to the retrospective and multicentric design of the study. This may compromise results, and the reported patient outcomes may therefore be biased. Further, the heterogeneity of the patient cohort, which included both AAA and PAU patients, limits the general comparability to the current literature. Further, the reported long-term outcomes may be considered with care, given the significant number of patients lost during early follow-up. Consequently, the reported follow-up outcome may be heavily biased. Next, this is a single-armed study without control groups for different treatments or stent grafts. Lastly, the small cohort limits the overall generalizability of the reported results.

## 5. Conclusion

Narrow aortic bifurcations remain challenging. EVAR using the AFX stent graft in very narrow aortic bifurcations at a mean diameter of 15.8 mm is safe and generates a satisfying short- and long-term patient outcome. Specifically, the outcomes may be superior in PAU when compared to AAA.

## Figures and Tables

**Table 1:** Patient demographics and comorbidities. Patient demographics and comorbidities are presented as mean ± standard deviation or absolute and relative frequencies (*n* (%)) for abdominal aortic aneurysm (AAA) and penetrating aortic ulcer (PAU) (*n* = 35).

Target	AAA (*n* = 17)		PAU (*n* = 18)		Total (*n* = 35)	
	Frequency distribution/mean	Percentage (%)/standard deviation	Frequency distribution/mean	Percentage (%)/standard deviation	Frequency distribution/mean	Percentage (%)/standard deviation
Gender (m : f)	13 : 4	76.5 : 23.5	14 : 4	77.8 : 22.2	27 : 8	77.1 : 22.9
ASA classification	II: 4/17 III: 7/17 IV: 6/17	II: 23.6 III: 41.1 IV: 35.3	II: 6/18 III: 10/18 IV: 2/18	II: 33.3 III: 55.6 IV: 11.1	II: 10/35 III: 17/35 IV: 8/35	II: 28.6 III: 48.6 IV: 22.8
Age (years)	71.2	6.6	75.8	7.6	73.8	7.2
PAOD	8/17	47.1	2/18	11.1	10/35	28.6
Prior interventions (CAD or PAOD)	5/17	29.4	3/18	16.7	8/35	22.8
Type 2 diabetes	5/17	29.4	0/18	0	5/35	14.3
Smoking history	8/17	47.1	3/18	16.7	11/35	31.4
Hypertension	17/17	100	16/18	88.9	33/35	94.3
Hypercholesterinemia	17/17	100	14/18	77.8	31/35	88.6
CAD	5/17	29.4	6/18	33.3	11/35	31.4
CKD (serum creatinine level > 1.5 mg/dl)	3/17	17.6	5/18	27.8	8/35	22.8
COPD	6/17	35.3	4/18	22.2	10/35	28.6

m = male; f = female; ASA = American Society of Anesthesiologists; PAOD = peripheral artery occlusive disease; CAD = coronary artery disease; COPD = chronic obstructive pulmonary disease; CKD = chronic kidney disease; mg/dl = milligrams per deciliter; *n* = number.

**Table 2:** Preoperative artery dimensions. Data is derived from angulation-adjusted measurements and is presented as mean ± standard deviation with min–max range for abdominal aortic aneurysm (AAA) and penetrating aortic ulcer (PAU). Student's *t*-test or Mann–Whitney *U* test was applied according to the Kolmogorov-Smirnov normality test to identify differences between AAA and PAU patients (*n* = 35).

Target	AAA (*n* = 17)		PAU (*n* = 18)*^Ψ^*		*p* value	Total (*n* = 35)	
	Mean ± standard deviation	Min–max range	Mean ± standard deviation	Min–max range		Mean ± standard deviation	Min–max range
Max aortic diameter (mm)	51.6 ± 9.5	34-72	36.1 ± 6.8	23-72	*p* < 0.05^∗^	44.0 ± 11.4	23-72
Aortic bifurcation diameter (mm)	16.2 ± 2.12	12-18	15.9 ± 2.2	11-18	*p* = 0.50	15.8 ± 2.2	11-18
Max CIA left diameter (mm)	12.8 ± 2.00	8-15	11.8 ± 2.1	7-17	*p* = 0.60	11.9 ± 2.2	7-17
Min CIA left diameter (mm)	9.8 ± 1.5	7-12	10.6 ± 1.9	6-13	*p* = 0.14	10.1 ± 2.5	6–13
Max CIA right diameter (mm)	12.9 ± 2.3	10-20	12.4 ± 2.7	8-21	*p* = 0.87	12.7 ± 2.5	8–21
Min CIA right diameter(mm)	9.6 ± 1.4	8-12	10.7 ± 2.1	6-15	*p* = 0.44	10.4 ± 1.9	6-15
CIA length right (mm)	50.1 ± 16	24-76	53.8 ± 15.3	31-80	*p* = 0.88	51.9 ± 16.0	24-80
CIA length left (mm)	50.8 ± 14.1	29-83	53.3 ± 15.3	26-80	*p* = 0.61	52.1 ± 14.6	26-83
Max EIA left diameter (mm)	8.3 ± 1.6	7-10	7.7 ± 1.2	5.5-10	*p* = 0.18	8.0 ± 1.9	5.5-10
Min EIA left diameter (mm)	7.9 ± 1.9	5.5-10	7.6 ± 2.3	3-9	*p* = 0.06	7.8 ± 1.8	3-10
Max EIA right diameter (mm)	9.2 ± 1.5	7-10	8.8 ± 1.8	5-10	*p* = 0.63	9.0 ± 1.6	5–10
Min EIA left diameter (mm)	8.2 ± 2.3	3-10	7.9 ± 1.9	4.5-9	*p* = 0.62	8.1 ± 2.2	3-10
Max infrarenal aortic neck diameter (mm)	28.4 ± 10.8	17-61	20.9 ± 2.8	17-26	*p* < 0.05^∗^	24.8 ± 8.2	17-61
Infrarenal neck length (mm)	36.2 ± 16.8	10-68	38.4 ± 17.8	15-80	*p* < 0.05^∗^	38.2 ± 24.2	10-80

CIA = common iliac artery; EIA = external iliac artery; min = minimum; max = maximum. *^Ψ^*Including one patient who was treated for a type Ib endoleak (EL) using the AFX stent graft (initial treatment for PAU).

**Table 3:** Key procedural data. Data is presented as mean ± standard deviation with the min–max range for abdominal aortic aneurysm (AAA) and penetrating aortic ulcer (PAU). Student's *t*-test or Mann–Whitney *U* test was applied according to the Kolmogorov-Smirnov normality test to identify differences between AAA and PAU patients (*n* = 35).

Target	AAA (*n* = 17)		PAU (*n* = 18)*^Ψ^*		*p* value	Total (*n* = 35)	
	Mean ± standard deviation	Min–max range	Mean ± standard deviation	Min–max range		Mean ± standard deviation	Min–max range
Procedure time (min)	127.0 ± 43.9	75-223	103.3 ± 35.2	54-171	*p* < 0.05∗	114.8 ± 39.9	54-223
Fluoroscopy time (min)	24.2 ± 7.8	9.5-40.0	13.5 ± 7.6	3.8–35.3	*p* < 0.05∗	19.9 ± 9.9	3.8-40
Contrast agent (ml)	43.9 ± 27.1	13-120	22.4 ± 14.5	15-70	*p* < 0.05∗	33.3 ± 22.5	13-120

^***Ψ***^Including one patient who was treated for a type Ib endoleak (EL) using the AFX stent graft (initial treatment for PAU).

## Data Availability

Data will be available upon reasonable request via e-mailing the corresponding author.
